# Intelligent diagnosis method for river and lake ecosystem health based on improved slime mold algorithm-optimized SVR

**DOI:** 10.1371/journal.pone.0340418

**Published:** 2026-01-21

**Authors:** Ran Chi, Yuewu Da, Weiying Li, Duo Wen

**Affiliations:** 1 College of Environmental Science and Engineering, Tongji University, Shanghai, China; 2 Wuxi Water Group Co., Ltd., Wuxi, China; Swedish Meteorological and Hydrological Institute, SWEDEN

## Abstract

In the diagnosis of river and lake ecosystems, there are complex nonlinear relationships among water quality parameters, and their dynamic change mechanisms are rather complicated. Traditional statistical analysis methods have limitations in providing precise assessment and timely early warning. To address the bottlenecks of traditional methods in accuracy, timeliness, and applicability, an intelligent diagnostic model based on improved slime mold algorithm-optimized support vector regression is proposed. This model improves its parameter optimization ability through dynamic weight strategy and adaptive search mechanism, and combines LightGBM feature selection to construct a combined model, effectively solving the problems of high-dimensional data modeling and dynamic adaptability. The experimental findings reveal that the optimized model improves indicators such as RMSE, MAE, and R^2^ compared to the comparative model. The RMSE is 0.031, the MAE is 0.021, and the R^2^ is 0.942. The prediction accuracy of the final proposed combination model is further optimized, with an RMSE of 0.022, an MAE of 0.016, and an R^2^ of 0.976. In addition, the average memory usage of the combined model is 120.5MB. The average sensitivity to outliers was 0.2, and the values were all better than those of the comparison models. At the same time, the prediction effects on pH value, dissolved oxygen, permanganate index, total phosphorus index, ammonia nitrogen index and chemical oxygen demand are relatively good. The research provides efficient and accurate methods for water quality prediction and ecosystem health diagnosis. The results show that the model proposed in the study has superior performance in the diagnosis of river and lake ecosystems and a good actual prediction effect. The intelligent diagnostic method proposed in the study enhances the ecological risk prevention and control capabilities of rivers and lakes, and promotes the digital transformation of water environment management.

## 1. Introduction

Rivers and lakes, as vital natural elements of the Earth, serve an indispensable function in maintaining biodiversity, purifying water quality, and regulating climate. However, with the swift advancement of industrialization and urbanization, river and lake ecosystems have faced unprecedented pressure, with water pollution being particularly prominent [1,2]. Accurately and efficiently predicting water quality parameters is key to achieving accurate assessment and early warning in the field of river and lake ecosystem health diagnosis. Water quality parameters, such as pH value, dissolved oxygen (DO), chemical oxygen demand, etc., are key indicators for evaluating the health status of rivers and lakes. These parameters not only reflect the physical and chemical characteristics of water bodies, but also directly affect the viability of water-dwelling creatures and the balance of ecosystems [3,4]. Therefore, accurate prediction and evaluation of these water quality parameters hold substantial importance for the oversight of river and lake ecosystems. At present, water quality assessment predominantly depends on consistent in-situ sampling and subsequent lab testing. Conventional approaches are both lengthy and demanding in terms of labor, and they fall short in delivering data in real time [5,6]. Although the method of on-site sampling and laboratory analysis can provide relatively accurate measurement results of water quality parameters, it is time-consuming and labor-intensive, and cannot achieve real-time monitoring of river and lake ecosystems. In practical operation, due to the limitations of human resources, material resources and time, the sampling frequency is often low, which leads to the inability to capture the dynamic changes of water quality parameters in a timely manner and makes it difficult to meet the timeliness requirements of river and lake ecosystem health diagnosis. Secondly, traditional statistical analysis methods have obvious deficiencies when dealing with the complex nonlinear relationships and dynamic change mechanisms of river and lake ecosystems. There are complex interactions and nonlinear coupling relationships among water quality parameters. However, traditional statistical methods usually assume that there are linear relationships among the data, which makes it difficult for them to accurately reflect the actual situation during modeling and prediction, resulting in low prediction accuracy. With the popularization of deep learning technology, the method of predicting various indicators of river and lake ecosystems based on neural networks for intelligent diagnosis has gradually become a major research hotspot.

To solve the above problems, the study proposes an intelligent diagnosis model for river and lake ecosystems based on the improved Slime Mould Algorithm (SMA) to optimize Support Vector Regression (SVR). This model enhances parameter optimization capabilities through dynamic weight strategies and adaptive search mechanisms, and combines LightGBM feature selection to construct a combined model, effectively addressing the issues of high-dimensional data modeling and dynamic adaptability. The innovations of this study are: (1) Combining the feature selection method of the lightweight Gradient Boosting Machine (LightGBM) with the improved slime mold algorithm not only improves the accuracy of the model’s prediction of water quality parameters, but also reduces the complexity and computational cost of the model by reducing the number of features. (2) The introduced dynamic weight strategy and adaptive search mechanism enable the algorithm to flexibly handle complex solution Spaces and enhance the efficiency at different search stages.

## 2. Literature review

At present, scholars have used neural network models to diagnose ecosystem health. C. Liu et al. introduced a comprehensive evaluation model based on expert knowledge and artificial neural networks to address the complex situation of river ecosystem health assessment. In the case study, the method achieved an evaluation result of a mean absolute error (MAE) of 4.78 in its application in China, while the traditional multiple linear regression baseline model had an MAE of 8.653, further demonstrating the effectiveness and superiority of the model [7]. Y. S. Kwon et al. proposed an evaluation model based on deep neural networks and nonlinear data mapping to assess the health status of freshwater ecosystems. The research outcomes indicated that the model exhibited the best performance in evaluating eutrophication diatom index, benthic macroinvertebrate index, and fish evaluation index, with root mean square errors (RMSEs) of 0.592 and 17.249 [8]. H. Meng et al. introduced a water quality classification model grounded in convolutional neural networks (CNNs) to handle the current situation of water quality classification in inland lakes of Dianchi Lake. The research results indicated that from Nov. 2020 to Apr. 2023, the overall water quality of Dianchi Lake improved, with an average proportion of 1.24% for water quality grade A (good water quality) and 84.28% for water quality grade B (slightly polluted water quality). Since Oct. 2022, the water quality of Dianchi Lake remained stable at level B, accounting for an average of 98.17% [9]. G. Gandhimathi et al. introduced a comprehensive water quality monitoring model that combines CNNs and gated loop units to handle the current status of river water quality monitoring. The research results indicated that the model performed well in monitoring key water quality parameters such as pollution level, turbidity, pH value, temperature, and DO. It can offer instant data gathering and evaluation, allowing relevant parties to promptly identify alterations in water quality. Compared with traditional methods and other advanced methods, this model had significant advantages in validation accuracy [10]. Q. Guo et al. proposed a hybrid deep learning model (CNN-LSTM) that combines convolutional neural network (CNN) and Long short-term memory network (LSTM) in response to the current sensitivity of urban forest ecosystems to temperature changes under the background of climate change, for predicting the monthly average and extreme atmospheric temperatures in Zhengzhou City, China. The results show that this model outperforms traditional models such as artificial neural Network (ANN), CNN, LSTM and CNN-GRU in terms of temperature prediction accuracy. It can capture the trend of temperature changes more accurately, providing a scientific basis for forest ecosystem management and response to climate change [11]. J. M. Souza et al. proposed a comprehensive energy system planning model based on an improved multi-objective particle swarm optimization (MOPSO) algorithm in response to the current situation where traditional energy systems are difficult to simultaneously meet the requirements of low carbon emissions and optimal economic benefits, aiming to collaboratively optimize system investment costs, operating costs, and carbon emissions. The results show that this model can reduce the total system cost by 8.7% and carbon emissions by 23.5% while ensuring the reliability of energy supply, providing a feasible path for the low-carbon economic planning of city-level integrated energy systems [12].

Although existing research has made certain progress in the prediction of water quality parameters, there are still some problems that have not been fully solved. Firstly, when dealing with high-dimensional data, the existing models have limited capabilities in feature selection and parameter optimization, resulting in high model complexity and high computational costs. Secondly, the existing models have deficiencies in dynamic adaptability and are difficult to effectively deal with the dynamic changes of water quality parameters. In addition, the existing models perform poorly in terms of robustness and have insufficient processing capabilities for outliers and noisy data, which affects the stability and reliability of the models. Based on this research, a smart diagnosis method for river and lake ecosystems is proposed, which optimizes support vector regression (SVR) using the improved slime mold algorithm (SMA). The research aims to develop a new intelligent diagnostic model to achieve accurate assessment and early warning of the health of river and lake ecosystems.

## 3. Methods and materials

### 3.1. Construction of SVR model based on SMA

#### 3.1.1. Algorithm principle and model parameters.

Traditional diagnostic methods rely heavily on statistical analysis, but the water quality parameters of rivers and lakes exhibit complex nonlinear coupling relationships and dynamic evolution mechanisms, which limits the accuracy, timeliness, and applicability of traditional methods. To overcome this bottleneck, the idea of introducing SMA optimization to support SVR is studied. SMA is an emerging swarm intelligence optimization algorithm, which is inspired by the foraging behavior of slime molds in nature. Slime molds are single-celled organisms that exhibit complex behavioral patterns during foraging, including the formation of network structures, secretion of signaling chemicals, and group coordination. These behaviors enable slime molds to efficiently search for and consume food resources in a vast environment. In the SMA algorithm, each solution in the algorithm represents the position of the slime mold in the solution space, which corresponds to a potential solution to the optimization problem. The fitness of each solution is determined by the objective function, which assesses the quality of the solution, that is, the degree to which the solution ADAPTS to the optimization problem. The algorithm simulates the group behavior of slime molds, including the interaction between solutions and information transmission. This is usually achieved by simulating the chemical substances secreted by slime molds, and the interaction between solutions can guide the algorithm to search for better solution space regions. The SMA algorithm conducts search and optimization by simulating the foraging behavior of slime molds, which includes global and local search of the solution space, as well as the update and improvement of solutions.

#### 3.1.2. An improved method of SMA based on dynamic weight strategy.

In the diagnosis of ecosystem health in rivers and lakes, although traditional SMAs can optimize the parameters of SVR models to a certain extent, they still have some limitations when facing complex water quality parameter prediction problems, such as insufficient global search ability, slow convergence speed, and inflexible parameter tuning [13,14]. Based on this research, an improved SMA is proposed, which improves the performance of the algorithm by introducing dynamic weight strategy and adaptive search mechanism. Firstly, the dynamic weighting strategy can dynamically adjust the weights based on the fitness of the solution, enhancing the collaborative optimization ability of the group. Specifically, the weights of each solution are updated after each iteration, and the computation for weight updates is shown in formula (1).


$$\omega_{i}^{\left(t+1\right)}=\frac{f(X_{i}^{\left(t\right)})}{\sum j=1Nf(X_{j}^{\left(t\right)})}$$ (1)

In formula (1), $\omega_{i}^{\left(t+1\right)}$ represents the weight of the *i*th solution in the *t* + 1th iteration, $f(X_{i}^{\left(t\right)})$ denotes the fitness of the *i*th solution in the *t*th iteration, *N* indicates the total number of solutions, and Σ represents summation [15]. To further enhance the effect of dynamic weights, the study introduces fitness normalization and a weight adjustment factor. In the intelligent diagnosis of river-lake ecosystem health, the range of fitness values can be extremely wide, which may lead to instability issues during weight calculation [16,17]. Therefore, the fitness values are normalized, and the relevant computation is presented in formula (2).


$$f_{norm}(X_{i}^{\left(t\right)})=\frac{f(X_{i}^{\left(t\right)}-\min_{j}(X_{i}^{\left(t\right)}))}{\max_{j}X_{i}^{\left(t\right)}-\min_{j}X_{i}^{\left(t\right)}}$$ (2)

In formula (2), $f_{norm}(X_{i}^{\left(t\right)})$ represents the normalized weight value, while $\min_{j}(X_{i}^{\left(t\right)})$ and $\max_{j}(X_{i}^{\left(t\right)})$ respectively denote the min and max fitness values of all solutions in the current iteration [18]. To further enhance the dynamic adjustment capability of weights, the study introduces a weight adjustment factor λ, which is utilized to adjust the weights. The relevant computation for weight adjustment is shown in formula (3).


$$\omega_{i}^{\left(t+1\right)}=\lambda \cdot \frac{f_{norm}(X_{i}^{\left(t\right)})}{\sum j=1Nf_{norm}(X_{j}^{\left(t\right)})}$$ (3)

In formula (3), the value of λ changes dynamically with the iteration number t [19]. Its specific computation is shown in formula (4).


$$\lambda =\lambda_{\max}-\frac{(\lambda_{\max}-\lambda_{\min})\cdot t}{T}$$ (4)

In formula (4), $\lambda_{\max}$ and $\lambda_{\min}$ are the max and min values of the weight adjustment factor, and T denotes the max iteration count [20]. The dynamic weight strategy enables the weight of each solution to be dynamically adjusted according to its fitness, thereby enhancing the collaborative optimization ability of the group. In the global search stage, solutions with higher fitness will receive higher weights, guiding the group to quickly approach the optimal solution. Meanwhile, the dynamic weight strategy further enhances the accuracy of parameter optimization through fitness normalization and weight adjustment factors.

#### 3.1.3. An improved method of SMA algorithm based on adaptive search mechanism.

**Fig 1:**
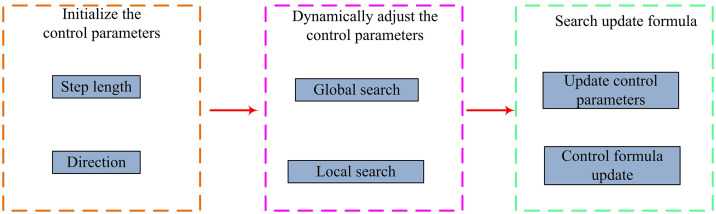
Implementation steps of adaptive search mechanism.

Considering that the search mechanism of the traditional SMA is relatively fixed and lacks the ability to adaptively modify the search step length and orientation based on the ongoing search condition, the study further introduces an adaptive search mechanism. This mechanism fosters the algorithm’s ability to balance between global and local searches by dynamically adjusting the step length and orientation. Specifically, the adaptive search mechanism adjusts the search step length and orientation by introducing control parameters α, β, and γ. The implementation steps of the adaptive search mechanism are shown in Fig 1.

In Fig 1, the adaptive search mechanism first initializes the control parameters, which include the upper and lower limits for regulating the search step size, the upper and lower bounds for determining the search direction, as well as the max and min values of the adjustment factor. Subsequently, it dynamically adjusts these control parameters. During the global search phase, the search step size α is relatively large, while β and γ are more randomly set to rapidly explore the solution space. In the local search phase, the search step size α is smaller, and β and γ are set more randomly but with greater precision to refine the solutions [21]. The computation for the search step size is shown in formula (5).


$$\alpha (t)=\alpha_{\max}-\frac{(\alpha_{\max}-\alpha_{\min})\cdot t}{T}$$ (5)

Meanwhile, the computations for the control parameters β and γ are shown in formula (6) [22].


$$\left\{ \begin{array}{c} \beta (t)=\beta_{\max}-\frac{(\beta_{\max}-\beta_{\min})\cdot t}{T}\\ \gamma (t)=\gamma_{\max}-\frac{(\gamma_{\max}-\gamma_{\min})\cdot t}{T} \end{array}\right.$$ (6)

After dynamically adjusting the control parameters, the search update formula is immediately applied. That is, in each iteration, the location of each solution is updated according to the current control parameters. The computation for the search update is presented in formula (7).


$$X_{i}^{\left(t+1\right)}=X_{i}^{\left(t\right)}+\alpha (t)\cdot sgn(X_{best}^{\left(t\right)}-X_{i}^{\left(t\right)})\cdot (|X_{best}^{\left(t\right)}-X_{i}^{\left(t\right)}\cdot \beta (t){)}^{\gamma (t)}$$ (7)

**Fig 2:**
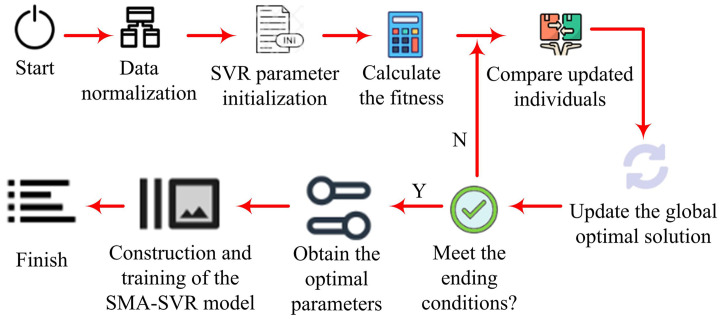
Flowchart of SMA optimized SVR model.

In formula (7), $X_{best}^{\left(t\right)}$ represents the optimal solution in the *t*th iteration, it is the one with the best fitness among all individuals [23]. In each iteration, the algorithm tracks and updates this optimal solution to guide the search process. sgn denotes the sign function. In an optimization algorithm, for each $X_{i}^{\left(t\right)}$, as the iterative process progresses, each individual will update its position according to the algorithm’s rules in order to find a better solution. For hyperparameters such as $\alpha_{\max}$, $\beta_{\max}$, $\gamma_{\max}$, etc., the research conducts a sensitivity analysis on them, that is, by fixing other parameters respectively and changing one parameter, the changes in model performance are observed. The research simultaneously changes the combination of multiple parameters to observe the variations in model performance. Ultimately, the dynamic adjustment of α(t) helps the algorithm quickly explore the solution space in the global search stage, thereby accurately locating the optimal solution in the local search stage. By controlling the search direction and step size, β(t) and γ(t) help enhance the algorithm’s ability to balance global search and local search at different search stages. Formula (7) optimizes the search of the solution space by simulating the foraging behavior of slime molds in nature and their stretching and contracting behaviors during the process of seeking food sources. Standard SMA usually adopts a fixed weight or a simple weight update mechanism, which cannot fully adapt to the dynamic changes of the solution space. In the research, by introducing the dynamic weight strategy, the integrable weight method can more flexibly adjust the search direction and step size according to the fitness of the solution in the current iteration, thereby improving the global search of the algorithm. The specific process of optimizing the SVR model based on the improved SMA is presented in Fig 2.

In Fig 2, during the construction of the SVR model grounded in the improved SMA, the parameter space of the SVR model includes the regularization parameter C, the kernel function parameter r, and the error tolerance ε. Therefore, the architecture for optimizing the SVR model based on the SMA is described as follows. Firstly, there is the initialization process, where a set of initial solutions is randomly generated, with each solution representing a combination of parameters (C,r,ε) for the SVR model. Secondly, there is fitness evaluation, which involves training the SVR model using the parameter combinations corresponding to each solution and simultaneously calculating the model’s fitness on the validation set. In this study, the fitness is measured by the RMSE, and its computation is shown in formula (8).


$$f(X_{i})=RMSE(X_{i})=\sqrt{\frac{\text{1}}{n}\sum_{j=1}^{n}{\left(y_{j}-y_{j}^{\sim }\right)}^{2}}$$ (8)

In formula (8), n is the number of samples in the validation set, $y_{j}$ denotes the actual value (AV), and $y_{j}^{\sim }$ indicates the predicted value (PV) [24]. Subsequently, based on the results of the fitness evaluation, the weights of each solution are updated. Finally, the locations of each solution are updated according to the dynamic weights and the adaptive mechanism. The aforementioned steps are repeated until the max iteration count is reached or the convergence criteria are satisfied. After the parameter optimization is completed, the SVR model is trained using the optimal parameter combination and validated on an independent test set to assess the generalization capability of this optimized model.

### 3.2. Construction of water quality prediction model grounded in Improved SVR and LightGBM

#### 3.2.1. Overview of ISVR and LightGBM.

**Fig 3:**
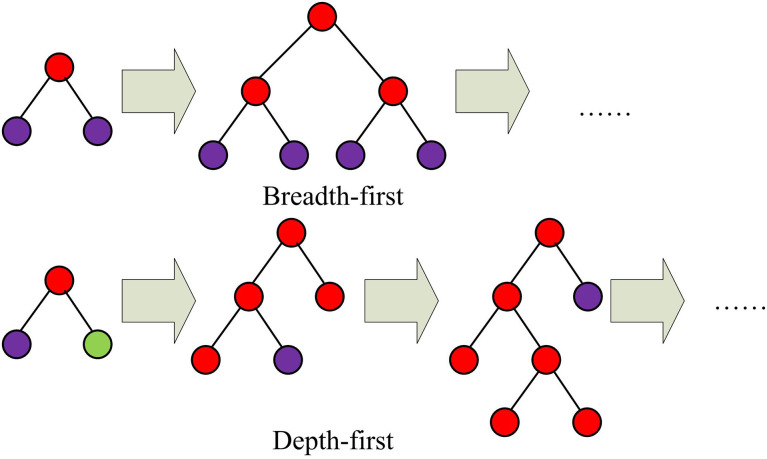
Schematic diagram of the LightGBM principle.

In the ISVR model constructed in the above-mentioned research, it can accurately predict water quality parameters. To further enhance the accuracy and efficiency of prediction, it is necessary to consider the optimization of the model and the feature selection strategy. Therefore, this study combines the improved ISVR model and LightGBM feature selection technology to construct a more efficient and accurate water quality prediction model. This method not only enhances the model’s response ability to changes in water quality parameters, but also reduces unnecessary computational burdens through feature selection, thereby achieving better prediction performance. In the diagnosis of river and lake ecosystem health, the prediction of water quality parameters not only relies on optimized model parameters, but also requires effective feature selection methods to process high-dimensional data [25]. High dimensional data often includes numerous superfluous and disruptive features, which can reduce the predictive performance of the model and increase computational costs. To overcome this problem, a highly efficient water quality prediction model is constructed by combining the ISVR model with LightGBM feature selection. The original feature set contains 30 features, but after feature selection, only 6 features remain [26]. The selected features include pH, DO, Permanganate Index, Total Phosphorus, Ammonia Nitrogen and Chemical Oxygen Demand. Feature selection can reduce the noise interference of the model by eliminating features that are irrelevant or have a minor impact on water quality parameters, thereby improving the prediction accuracy of specific water quality parameters [27]. LightGBM is an efficient gradient boosting framework algorithm that performs well when dealing with large-scale datasets, featuring high speed and high efficiency. Among them, LightGBM uses the histogram algorithm to optimize the selection of split points during the training process. This method divides the value range of continuous features into several histogram intervals, thereby avoiding the search for split points for each possible value of each feature and significantly accelerating the training speed. Meanwhile, LightGBM supports parallel learning, allowing models to be trained in parallel on multi-core cpus and also enabling the use of Gpus to accelerate the training process, further enhancing the training speed. In addition, LightGBM provides feature importance assessment, which can help identify the features that have the greatest impact on model predictions, thereby achieving feature selection. Its principle is shown in Fig 3.

In Fig 3, LightGBM is capable of automatically processing feature importance in high-dimensional data and selecting key features through feature importance scoring. Specifically, the computation of the feature importance score is presented in formula (9).


$$I(F_{i})=\sum_{j=1}^{Q}Gain(F_{i},Q_{j})$$ (9)

In formula (9), $I(F_{i})$ is the importance score of feature $F_{i}$, Q denotes the total number of trees, and $Gain(F_{i},Q_{j})$ indicates the gain value of feature $F_{i}$ in the j th tree [28]. The computation for the gain value is shown in formula (10).


$$Gain(F_{c},Q_{d})=\frac{\text{1}}{\text{2}}\left(\frac{(\sum c\in J_{L}g_{c}{)}^{2}}{\sum c\in J_{L}h_{c}+\zeta }+\frac{(\sum c\in J_{R}g_{c}{)}^{2}}{\sum c\in J_{R}h_{c}+\zeta }-\frac{(\sum c\in Jg_{c}{)}^{2}}{\sum c\in Jh_{c}+\zeta }\right)-\eta$$ (10)

In formula (10), $J_{L}$ and $J_{R}$ represent the sample index sets of the left and right subtrees after splitting, respectively. J denotes the sample index set before splitting. $g_{c}$ and $h_{c}$ represent the first- and second-order derivatives of sample c. ζ indicates the regularization term. η represents the penalty term for the amount of leaf nodes [29].

#### 3.2.2. The optimization method of LightGBM based on C_PSO.

To optimize the performance of LightGBM, the study introduces a Chaotic Particle Swarm Optimization (C_PSO) algorithm to optimize the hyperparameters of LightGBM. This algorithm incorporates chaotic mapping into the traditional PSO algorithm to enhance population diversity, thereby improving the algorithm’s global search capability and preventing it from getting trapped in local optimal solutions [30]. Because different datasets have different characteristics, including the number of features, feature types, data distribution, etc. In datasets with a large number of features, complex feature types, and uneven data distribution, the dynamic weight strategy and adaptive search mechanism of the model can more effectively optimize parameters and improve the prediction performance of the model. For datasets with a small number of features or uniform data distribution, complex feature selection and parameter optimization processes may not be necessary. In this case, a simpler model or the default parameter Settings of LightGBM can be used to complete the prediction. By optimizing hyperparameters through the C_PSO algorithm, better parameter configurations can be found for specific datasets, thereby improving the prediction performance of the model [31]. The hyperparameter space of LightGBM is extremely complex, containing multiple continuous and discrete parameters. The C_PSO algorithm can effectively explore this parameter space and find the optimal or near-optimal combination of hyperparameters. Compared with traditional methods such as grid search or random search, the C_PSO algorithm usually requires fewer evaluation times while finding a satisfactory solution, which makes it more computationally efficient. In the C_PSO algorithm, the computation for the location of each particle is shown in formula (11).


$$x_{i}^{\left(t+1\right)}=x_{i}^{\left(t\right)}+v_{i}^{\left(t+1\right)}$$ (11)

In formula (11), $x_{i}^{\left(t\right)}$ represents the location of the *i*th particle in the *t*th iteration, and $v_{i}^{\left(t+1\right)}$ represents the velocity of the *i*th particle in the *t*th iteration [32]. The relevant computations are shown in formula (12).


$$v_{i}^{\left(t+1\right)}=e\cdot v_{i}^{\left(t\right)}+c_{1}\cdot r_{1}\cdot (p_{i}^{\left(t\right)}-x_{i}^{\left(t\right)})+c_{2}\cdot r_{2}\cdot (g^{\left(t\right)}-x_{i}^{\left(t\right)})$$ (12)

In formula (12), e is the inertia weight, $c_{1}$ and $c_{2}$ represent the learning factors, $r_{1}$ and $r_{2}$ represent random numbers with values between [0,1], $p_{i}^{\left(t\right)}$ represents the individual optimal location of the *i*th particle in the *t*th iteration, and $g^{\left(t\right)}$ represents the global optimal location in the *t*th iteration [33]. The introduction of chaotic mapping can increase the randomness of particles and enable them to conduct a broader exploration within the search area. The computation of chaotic mapping is presented in formula (13).


$$x_{i+1}=\mu \cdot x_{i}\cdot (1-x_{i})$$ (13)

In formula (13), μ represents the chaotic mapping parameter [34]. It can increase the randomness of particle position update in the particle swarm optimization algorithm, thereby improving the performance of the algorithm.

#### 3.2.3. Model training and diagnosis process.

After completing feature selection based on the improved LightGBM, the selected key features are utilized to train the ISVR model. The construction process of the ISVR-LightCBM model can be characterized as below. Firstly, the original data undergoes standardization processing, and the relevant computation is presented in formula (14).


$$X_{norm}=\frac{X-M(X)}{R(X)}$$ (14)

**Fig 4:**
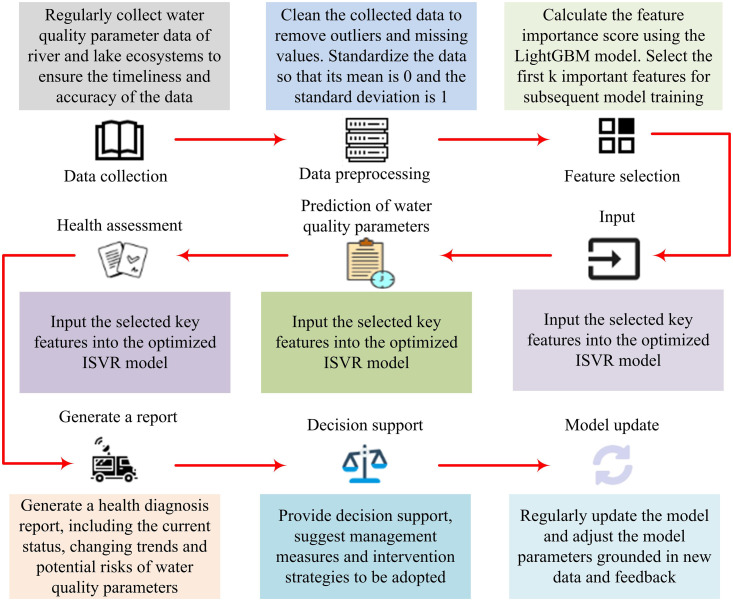
Intelligent diagnosis process for the health of river and lake ecosystems.

In formula (14), $X_{norm}$ is the standardized data, M(X) denotes the median of the data, and R(X) indicates the interquartile range of the data [35]. Subsequently, LightGBM is employed to select key features by training the LightGBM model to calculate the importance score of each feature and making selections accordingly [36,37]. Finally, the specified key features are chosen to train the ISVR model, and the model’s performance is validated on an independent test set. The intelligent diagnosis process for the health of river-lake ecosystems is illustrated in Fig 4.

In Fig 4, the data collection process involves regularly gathering water quality parameter data from river-lake ecosystems to guarantee the timeliness and accuracy of the data. Data preprocessing entails cleaning the collected data by removing outliers and missing values, as well as normalizing it to achieve a mean of 0 and a standard deviation of 1 [38,39]. The feature selection process utilizes the LightGBM model to calculate feature importance scores, and the important features are then used for subsequent model training. The selected key features are input into the optimized ISVR model. The ISVR model is employed to predict water quality parameters and generate prediction results. Based on these prediction results, the health status of the river-lake ecosystem is assessed, and a health threshold is set. When the predicted water quality parameters exceed this threshold, an early warning signal is issued [40,41]. Following the assessment, a health diagnosis report is generated, which includes the current status, trends, and potential risks of water quality parameters [42,43]. Based on the health diagnosis report, decision support is provided, recommending management measures and intervention strategies. Meanwhile, the diagnosis results and recommendations are fed back to environmental management departments for formulating and implementing river-lake ecological protection plans [44,45]. Finally, the model is regularly updated, with model parameters adjusted according to new data and feedback to ensure the model’s ongoing effectiveness [46].

## 4. Results

### 4.1. Analysis based on ISVR-LightGBM model

To confirm the effectiveness of the introduced intelligent diagnostic model based on improved SMA optimized SVR combined with LightGBM feature selection. To verify the effectiveness of the proposed intelligent diagnostic model based on the improved SMA for optimizing SVR combined with LightGBM feature selection, the study took Poyang Lake in the middle and lower reaches of the Yangtze River in China as the research object. Poyang Lake is located in the northern part of Jiangxi Province, on the south bank of the Yangtze River. It is the largest freshwater lake in China and also an important lake in the Yangtze River Basin in terms of water flow, throughput and seasonality. The time span for data collection was from January 2024 to December 2024. Among them, the sampling frequency for pH was once a day, for DO once every six hours, for chemical oxygen demand once a week, for total phosphorus once a month, and for ammonia nitrogen once every two weeks. During the data collection period, different sampling frequencies were adopted according to different water quality parameters and monitoring requirements. The dataset contains a total of 1080 samples. The dataset was divided into the training set, validation set and test set in sequence at a ratio of 7:2:1. For the collected data, the study first cleaned the raw data collected to remove outliers and missing values. Secondly, the cleaned data was standardized to make its mean 0 and standard deviation 1, thereby eliminating the differences in water quality parameters caused by range and dimension. To prevent data leakage, when researching the use of sliding window technology, it should be ensured that the data within each window only contained data prior to the current time point. At the same time, when creating lagging features, it should be ensured that the calculation of lagging features does not introduce future information; In addition, the study divided the data in chronological order to ensure that the data in the training set was earlier than that in the test set. Table 1 displays the setup of the experimental environment and the configurations of model parameters.

**Table 1:** Experimental environment and model parameter settings.

Component	Specification	Parameter (C_PSO)	Value	Parameter (ISVR)	Value	Parameter (LightGBM)	Value
Processor	Intel Core i7-9700K	Kernel	Radial Basis Function (RBF)	Population size	50	Number of trees	100
RAM	16GB DDR4	Regularization parameter (C)	1	Max iterations	100	Learning rate	0.1
GPU	NVIDIA GeForce GTX 1080 Ti	Kernel parameter	0.1	α_max	1	Max depth	5
Storage	1TB SSD	Epsilon	0.1	α_min	0.1	Subsample	0.8
Operating System	Windows 10 Pro	/	/	β_max	1	Colsample_bytree	0.8
Programming Language	Python 3.8	/	/	β_min	0.5	/	/
Libraries	Scikit-learn 0.24.2, LightGBM 3.3.2	/	/	γ_max	1	/	/
/	/	/	/	γ_min	0.5	/	/

In Table 1, the sample size of 50 was based on the results of grid search or random search, and the maximum number of iterations of 100 was to avoid overfitting while ensuring that the model had sufficient iterations to converge. The kernel parameter was 0.1, which was the best or closest value found in the grid search. The research adopted the method of grid search. After defining the parameter network, an SVR model was trained for each Kernel parameter. Through error evaluation, the model performance was finally determined to be optimal when the Kernel parameter was 0.1. For the optimization of hyperparameters, the research adopted two methods, namely grid search and random search, to explore the hyperparameter space. For each set of hyperparameters, the model was first trained and its performance was evaluated on the validation set. Then the study recorded the performance of each group of hyperparameters and select the best-performing combination. After the grid search, the study conducted a random search to further explore the hyperparameter space. A certain number of hyperparameter combinations were randomly generated, and the performance of each group was evaluated. Combining the results of grid search and random search, the study selected the combination of hyperparameters that performed best on the validation set. These hyperparameters were used for the training of the final model. To prevent overfitting of the model, the study tested different regularization strengths ranging from 0.01 to 1.0 and found that when the regularization coefficient was 0.1, the generalization ability of the model could be improved. Meanwhile, the maximum depth was another important hyperparameter that controlled the complexity of the model. In this study, the study analyzed different depth settings from 3 to 6 and found that when the maximum depth was 4, the model had no overfitting and could capture complex patterns. Based on the experimental environment and parameter settings presented in Table 1, a self-made dataset was utilized, all instruments were calibrated on-site before sampling to ensure the accuracy of the data. Meanwhile, each sampling point was sampled three times repeatedly, and the average value was taken as the final data. Outliers and missing values were removed from the collected data to ensure the integrity and reliability of the data. Meanwhile, descriptive statistical analysis was conducted on all parameters, including mean, standard deviation, minimum value, maximum value and median, etc. For specific parameters, pH, as an indicator of the acidity or alkalinity of water bodies, had a direct impact on the survival of aquatic organisms and the quality of water. DO reflected the self-purification capacity and biological activity of water bodies and was an important parameter for evaluating water quality. Permanganate index: As an indicator of organic pollution, the permanganate index could reflect the total amount of organic matter in water bodies. The study also selected CNN, Multilayer Perceptron (MLP), and Long Short-Term Memory (LSTM) for comparative experiments. To reflect the fairness of the comparison, the data preprocessing methods for all models were consistent, including normalization, standardization, and handling of missing values, etc. CNN processed through regularization techniques and the Adam optimizer; MLP attempted different numbers of hidden layers and the number of neurons in each layer, and optimized them using regularization techniques. LSTM optimized the number of LSTMS, the length of the input sequence, and the parameters of the gating mechanism.

**Fig 5:**
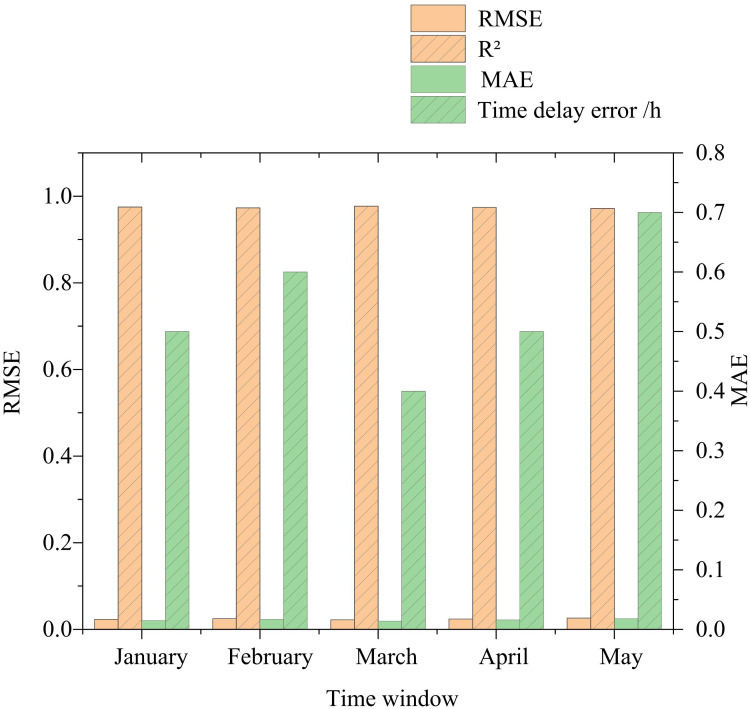
Water quality prediction results at different time Windows.

In Fig 5, the model’s performance was relatively stable at different time Windows, with small fluctuation ranges of RMSE, MAE and R² values, indicating that the model could well adapt to the dynamic changes of water quality parameters and had strong dynamic adaptability. Meanwhile, the time delay error of the model was relatively small, averaging 0.55 hours, indicating that the model could quickly capture the changes in water quality parameters and provide timely information for the early warning and management of river and lake ecosystems. The error comparison of the test sets based on the ISVR optimization model and the control model is shown in Table 2.

**Table 2:** Comparison of error results in the test set.

Parallel experiment	Model	RMSE	MAE	R^2^
Parallel Experiment 1	ISVR	0.035	0.025	0.947
	MLP	0.049	0.036	0.920
	CNN	0.045	0.029	0.931
	LSTM	0.039	0.026	0.942
Parallel Experiment 2	ISVR	0.027	0.017	0.937
	MLP	0.041	0.028	0.914
	CNN	0.037	0.021	0.921
	LSTM	0.031	0.018	0.938

In Table 2, the RMSE and MAE of the ISVR model remain at relatively low levels, at 0.031 and 0.021 respectively, indicating that the ISVR model has high accuracy in predicting water quality parameters. Meanwhile, its R² value is close to 0.95, which means that the model can explain the changes in most water quality parameters, demonstrating its strong ability to capture the variation patterns of water quality parameters. The outstanding performance of the ISVR model may be attributed to its optimized algorithm and parameter Settings, which make it more effective in handling complex data. In contrast, the RMSE and MAE of the MLP model are relatively high, being 0.045 and 0.032 respectively, which indicates that its error in predicting water quality parameters is relatively large. Meanwhile, the R² value of the MLP model is 0.917. Although the value is still relatively high, compared with other models, its explanatory ability is slightly weaker. These performances of the MLP model may be related to its model structure and may not be as effective as other models when dealing with nonlinear relationships and high-dimensional data. Finally, the RMSE and MAE of the CNN and LSTM models lie between those of ISVR and MLP, demonstrating moderate predictive performance. Meanwhile, the R² value of the CNN model is 0.931, while the R² values of the LSTM model are 0.942 and 0.938 respectively, indicating that these two models have certain capabilities in explaining the changes of water quality parameters. The performance of CNN and LSTM models may be influenced by their specific structures, such as the advantages of CNN in handling spatial data and the capabilities of LSTM in dealing with time series data. The study further compared the errors on the training set, and the results are shown in Table 3.

**Table 3:** Comparison of training set error results.

Parallel Experiment	Model	RMSE	MAE	R^2^
Parallel Experiment 2	ISVR	0.030	0.020	0.950
	MLP	0.046	0.032	0.925
	CNN	0.040	0.025	0.931
	LSTM	0.033	0.022	0.945
Parallel Experiment 2	ISVR	0.025	0.015	0.940
	MLP	0.040	0.025	0.913
	CNN	0.032	0.022	0.921
	LSTM	0.030	0.016	0.935

**Fig 6:**
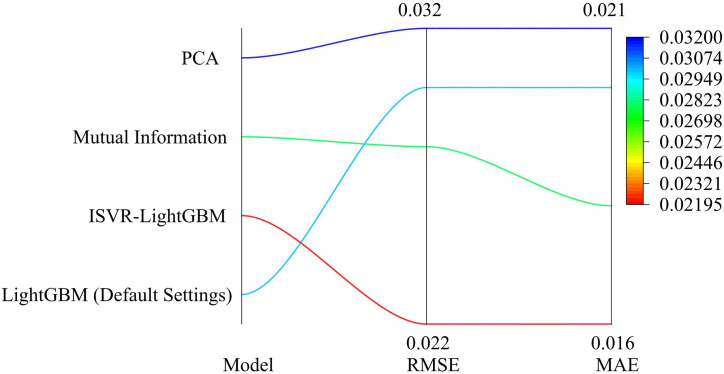
Comparison of error results.

In parallel Experiment 1, the ISVR model performed well on the training set, with an RMSE of 0.030, a MAE of 0.020, and an R² of 0.950. Compared with other models, it has lower errors and higher goodness of fit. In parallel Experiment 2, the ISVR model continued to maintain its advantage, with RMSE reduced to 0.025, MAE decreased to 0.015, and R² increased to 0.940, further demonstrating its efficient fitting ability on the training set. In contrast, the errors and fitting degrees of the other comparison models are relatively poor, indicating that the ISVR model can effectively learn and fit water quality parameter data during the training process, and has good parameter optimization capabilities and model fitting effects. The study further compared the capability of the ISVR-LightGBM model with that of the control models. The study further compared the default Settings, Mutual Information and PCA errors of the ISVR-LightGBM model with LightGBM, and the results are shown in Fig 6.

**Fig 7:**
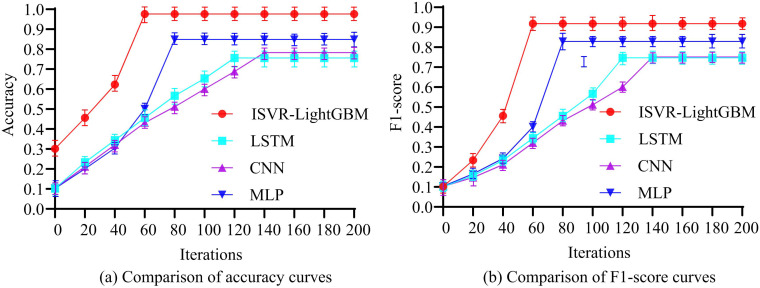
Comparison of the accuracy curve and the F1 score curve.

In Fig 6, the ISVR-LightGBM model outperformed the LightGBM model with default settings, the feature selection method based on mutual information, and the principal component analysis (PCA) method in both RMSE and MAE metrics. Specifically, the RMSE of the ISVR-LightGBM model was 0.022 and the MAE was 0.016. Both indicators were superior to the other three methods, indicating that the ISVR-LightGBM model had higher accuracy and reliability in predicting water quality parameters. In addition, compared with other feature selection methods, LightGBM feature selection performed better when dealing with high-dimensional data and could more effectively improve the prediction performance of the model. These results further verified the effectiveness and superiority of the ISVR-LightGBM model in the health diagnosis of river and lake ecosystems. Firstly, the comparison of accuracy and F1 score curves among the four models is shown in Fig 7.

Fig 7a indicates a comparison of the accuracy of four models. The outcomes revealed that the initial accuracy of the ISVR-LightGBM model was 0.301, and with the increase of iteration times, the accuracy rapidly improved, reaching 0.976 and stabilizing at 60 iterations. The LSTM model had the worst accuracy, with an initial accuracy of 0.102. As the iteration count increased, the accuracy gradually improved, reaching 0.756 and stabilizing at 120 iterations. Fig 7b shows the comparison of F1 scores among four models. The outcomes revealed that the F1 score of the ISVR-LightGBM model was notably higher than the other control models after convergence, with an initial F1 score of 0.102, reaching 0.918 at 60 iterations and tending to stabilize. The LSTM model had the worst F1 score, with an initial F1 score of 0.101. As the iteration count increased, the F1 score gradually improved, reaching 0.747 and stabilizing at 120 iterations. The study further compared the error values of the four models based on the test set, and the results are shown in Table 4.

**Table 4:** Comparison of training set error results.

Parallel experiment	Model	RMSE	MAE	R^2^
Parallel experiment 1	ISVR-LightGBM	0.024	0.018	0.984
	LSTM	0.037	0.024	0.943
	CNN	0.042	0.027	0.933
	MLP	0.047	0.034	0.923
Parallel experiment 2	ISVR-LightGBM	0.020	0.014	0.968
	LSTM	0.033	0.02	0.927
	CNN	0.038	0.023	0.917
	MLP	0.043	0.030	0.907

In Table 4, the average RMSE of the ISVR-LightGBM model was 0.022, and the MAE was 0.016, which was significantly lower than the control model. In addition, compared with ISVR, the error was further reduced, revealing that the model’s error in forecasting water quality parameters was further reduced, and the predicted results were closer to the AVs. In addition, the MLP model had the largest error, with an RMSE of 0.045 and an MAE of 0.032, revealing its weak ability to predict water quality parameters. The study further compared the error values of the four models based on the training set, and the results are shown in Table 5.

**Table 5:** Comparison of training set error results.

Parallel Experiment	Model	RMSE	MAE	R^2^
Parallel Experiment 2	ISVR-LightGBM	0.022	0.014	0.982
	LSTM	0.035	0.022	0.941
	CNN	0.043	0.025	0.938
	MLP	0.045	0.033	0.92
Parallel Experiment 2	ISVR-LightGBM	0.018	0.012	0.979
	LSTM	0.035	0.018	0.938
	CNN	0.035	0.022	0.927
	MLP	0.047	0.025	0.912

In parallel Experiment 1, the ISVR-LightGBM model demonstrated outstanding performance on the training set, with an RMSE of only 0.022, a MAE of 0.014, and an R² as high as 0.982. This indicates that the model has an excellent fitting effect on the training set and can capture the variation patterns of water quality parameters with extremely high precision. In parallel Experiment 2, the ISVR-LightGBM model continued to maintain its dominant position. RMSE was further reduced to 0.018, MAE was reduced to 0.012, and R² was increased to 0.979. This further proved the model’s efficient learning ability and strong fitting ability during the training process. In contrast, the errors and fitting degrees of the other comparison models are relatively poor. In summary, the ISVR-LightGBM model has achieved the lowest error and the highest goodness of fit on the training set. This is attributed to its combination of an improved slime mold algorithm to optimize SVR parameters and the use of LightGBM for feature selection, thus having significant advantages when dealing with high-dimensional data and complex nonlinear relationships. The study further compared the running time and computational complexity of the four models, and the results are shown in Table 6.

**Table 6:** Comparison of computing time and computational complexity.

Model	Training time/h	Inference time/milliseconds	Memory usage/GB	Computational complexity(FLOPs)
ISVR-LightGBM	0.5	0.05	2.3	1.2 × 10^9^
LSTM	1.8	0.21	3.1	5.0 × 10^9^
CNN	1.2	0.12	2.7	3.5 × 10^9^
MLP	0.3	0.03	1.3	8.0 × 10^8^

In Table 6, the MLP model had the shortest training time and the lowest computational complexity. This might be due to the relatively simple structure and fewer parameters of the MLP model. The ISVR-LightGBM model was at a medium level in terms of training time and computational complexity, but it combined the advantages of feature selection and might perform better on specific tasks. Due to its convolutional structure, the CNN model had advantages in tasks such as image processing, but its computational complexity was relatively high. Due to its ability to handle sequential data, the LSTM model had the longest training time and the highest computational complexity, which reflected its computational cost when dealing with time series data. The study further cross-validated and analyzed the error values of ISVR-LightGBM based on the 5-fold K value, and the results are shown in Table 7.

**Table 7:** Analysis of cross-validation results.

Fold numbe	RMSE	MAE	R²
1	0.023	0.015	0.975
2	0.025	0.017	0.973
3	0.022	0.014	0.977
4	0.024	0.016	0.974
5	0.026	0.018	0.972

**Fig 8:**
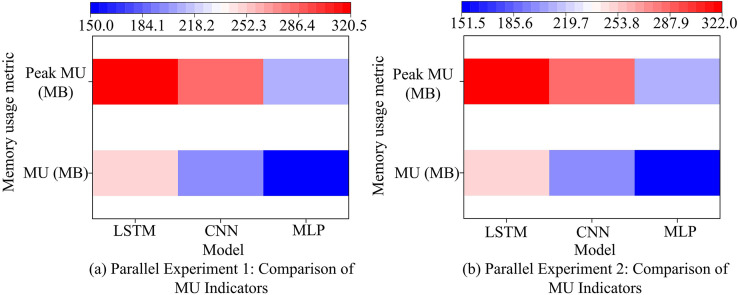
Comparison of MU metrics.

In Table 7, the average RMSE of the ISVR-LightGBM model was only 0.024, indicating that the error of the model in predicting water quality parameters was extremely small. The model could predict the changes in water quality parameters very accurately, and the prediction results were very close to the true values. Meanwhile, the MAE of the model was 0.016, further verifying the stability and generalization ability of the model on different data subsets. In addition, the average coefficient of determination of the model was 0.974, close to 1, indicating that the model could well explain the changes in water quality parameters. It could not only accurately predict water quality parameters but also capture the inherent laws of water quality parameter changes, providing a reliable basis for the health diagnosis of river and lake ecosystems. The study further compared the memory usage (MU) metrics of four models, and the results are shown in Fig 8.

**Fig 9:**
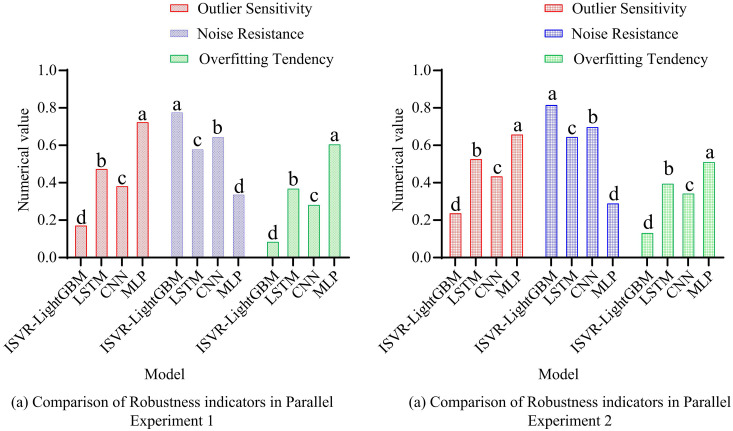
Comparison of model robustness indicators.

In the application of river and lake ecosystem health diagnosis, many environmental monitoring institutions may face the limitation of computing resources. Efficient memory usage can ensure that the application runs under limited hardware conditions, thus enabling more institutions to deploy and use this technology. Fig 8a indicates the comparison of MU indicators for the parallel experiment 1 model, and Fig 8b shows the comparison of MU indicators for the parallel experiment 2 model. The outcomes revealed that the average MU of the ISVR-LightGBM model was 120.5MB, which was significantly lower than the control model. This indicated that the model combined the improved SMA and LightGBM feature selection, and had high memory efficiency, rendering it appropriate for use in resource constrained environments. In addition, the average peak MU of ISVR-LightGBM was 180.7MB, indicating an increase in memory requirements during feature selection and parameter optimization processes. The traditional LSTM model had the highest MU and peak value, with values of 250.3MB and 320.5MB, indicating that the LSTM model required more memory to store model parameters and intermediate calculation results. Meanwhile, its memory requirements significantly increased when processing long sequence data. At the end of the study, the robustness indicators of four models were compared. Among them, Outlier Sensitivity measures the degree of sensitivity of the model to outliers in the dataset; Noise Resistance measures a model’s ability to maintain stable performance in the face of data noise. In the measurement program, a certain proportion of outliers and random noise were first added to the original data. Then, the model was trained using outliers. Finally, the performance of the model on datasets with and without outliers was compared. The outcomes are presented in Fig 9.

**Fig 10:**
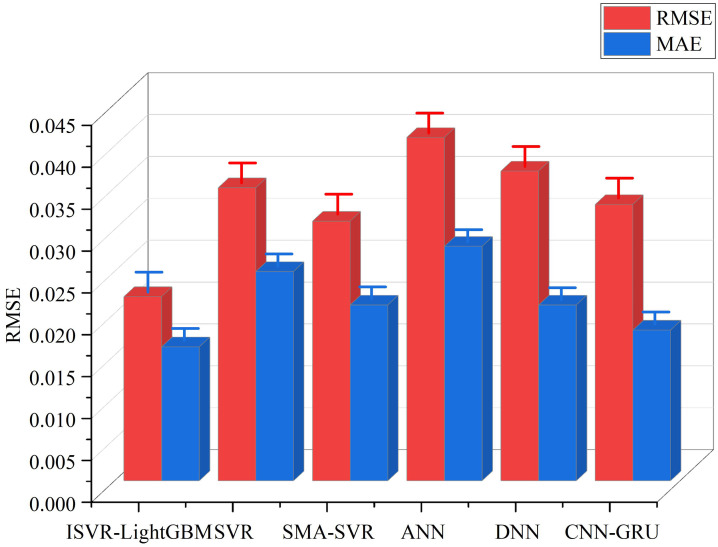
Comparison of error values of six models.

Fig 9 shows the robustness metrics of the four models in two parallel experiments. Among them, abcd represents the alphabetic notation in analysis of variance. When two groups have the same letters, it indicates a significant difference. In terms of outlier sensitivity, the ISVR-LightGBM model demonstrated the lowest outlier sensitivity in both experiments, with a value close to 0.2. This indicates that the model has a relatively low sensitivity to outliers in the data and can ignore these outliers to a certain extent, thereby maintaining the stability of the prediction. In contrast, the outlier sensitivity of other models is relatively high, especially the MLP model. This may imply that these models are more sensitive to outliers and are more susceptible to their influence, leading to a decline in prediction performance. In terms of anti-noise capability, the value of the SVR-LightGBM model is close to 0.8, indicating that this model can effectively resist noise interference in the data. The noise resistance of LSTM and CNN models comes second, while that of MLP models is the lowest. This might be related to its model structure, as MLP may be more susceptible to noise. In terms of overfitting tendency, the overfitting tendency value of the ISVR-LightGBM model is the lowest, close to 0.1, indicating that this model can effectively avoid overfitting during the training process and has good generalization ability. The overfitting tendency of other models is relatively high, especially MLP models. This may imply that these models are more likely to remember the details and noise of the training data during the training process rather than learn the general patterns of the data, thereby affecting the performance of the models on new data. The study further introduced SVR, SMA-SVR, ANN, DNN and CNN-GRU for comparative experiments, and the results are shown in Fig 10.

In Fig 10, when comparing the performance of six models, it was found that the ISVR-LightGBM model demonstrated advantages. Specifically, the RMSE of the ISVR-LightGBM model was only 0.022, and the MAE was 0.016. Both key indicators were the lowest among all models, indicating that the ISVR-LightGBM model had the smallest error in predicting water quality parameters, and the prediction results were the closest to the true values. For example, compared with the traditional SVR model, the RMSE value of ISVR-LightGBM was 0.013 lower and the MAE value was 0.009 lower. Compared with the SMA-SVR model, the RMSE value of ISVR-LightGBM was 0.009 lower and the MAE value was 0.005 lower. This result is consistent with that of Y. Zou et al. [47]. It proposed an improved LightGBM model to evaluate the spatial pattern machine driving factors of China’s terrestrial carbon sinks. In the results, the MAE of the improved LightGBN was reduced by 27.73%, and the RMSE was reduced by 24.20%. Meanwhile, the prediction error band after optimization was significantly narrowed, and the stability of the model was effectively enhanced. These numerical differences demonstrated the good performance of the ISVR-LightGBM model in terms of prediction accuracy. In addition, the ISVR-LightGBM model not only improved the prediction accuracy of the model by combining the improved SMA to optimize the SVR parameters and using LightGBM for feature selection, but also enhanced its processing ability for high-dimensional data and dynamic adaptability. This feature gave the ISVR-LightGBM model an advantage in the diagnosis of river and lake ecosystem health, and it could provide efficient and accurate methods for water quality prediction and ecosystem health assessment. At the end of the study, the contribution of each component of the model to the model performance was verified through ablation experiments, and the results are shown in Table 8.

**Table 8:** Results of the ablation experiment.

Model configuration	RMSE	MAE	R^2^
ISVR-LightGBM (Full Model)	0.022	0.016	0.976
ISVR (Without LightGBM)	0.027	0.017	0.937
SMA-SVR (Without Dynamic Weight)	0.031	0.021	0.942
SVR (Without Dynamic Weight and Adaptive Search)	0.035	0.025	0.947
ISVR-LightGBM (Without Adaptive Search)	0.024	0.018	0.974
ISVR-LightGBM (Without Dynamic Weight)	0.026	0.019	0.972

In Table 8, the three components of dynamic weight strategy, adaptive search mechanism and LightGBM feature selection all made significant contributions to the performance of the ISVR-LightGBM model. Specifically, the RMSE of the complete model was 0.022, the MAE was 0.016, and the R² reached 0.976, demonstrating the best performance. When LightGBM feature selection was removed, the RMSE of the model rose to 0.027, the MAE was 0.017, and the R² dropped to 0.937, indicating that LightGBM feature selection played an important role in processing high-dimensional data and improving prediction accuracy. After removing the dynamic weighting strategy, the RMSE of the model was 0.026, the MAE was 0.019, and the R² was 0.972, indicating that the dynamic weighting strategy was indispensable in improving the global search ability and the accuracy of parameter optimization. When both the dynamic weight strategy and the adaptive search mechanism were removed simultaneously, the RMSE of the model further rose to 0.035, the MAE was 0.025, and the R² was 0.947. This further proved that the synergy of these two components in the overall performance of the model was crucial. Furthermore, when only the adaptive search mechanism was removed, the RMSE of the model slightly increased to 0.024, the MAE was 0.018, and the R² was 0.974, indicating that the adaptive search mechanism also made certain contributions to improving the local search ability and dynamic adaptability of the model. In conclusion, the synergistic effect of these components enabled the ISVR-LightGBM model to achieve precision and reliability in predicting water quality parameters, enhancing the accuracy of health diagnosis for river and lake ecosystems.

### 4.2. Verification of actual effect of water quality prediction based on ISVR-LightGBM model

**Fig 11:**
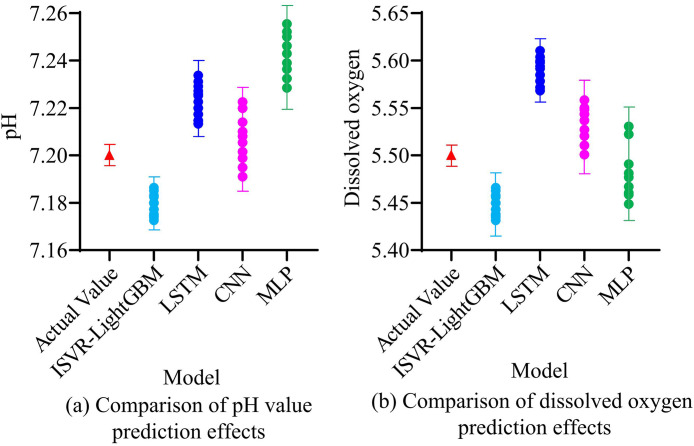
Comparison of the predictive effects of pH value and DO.

After validating the capability of the ISVR-LightGBM model, the research further compared the actual performance of water quality prediction grounded in four neural network models. Overall, the pH value measurement range was 6.5 to 8.5. The measurement range of DO was 5–10 milligrams per liter. The measurement range of chemical oxygen demand was 10–50 milligrams per liter. The measurement range of total nitrogen (TN) was 0.5 to 2.0 milligrams per liter. The measurement range of the permanganate index was 1–10 milligrams per liter. When both RMSE and MAE values were less than 5% to 10% of the measurement range of the corresponding variables, this error level was acceptable. The comparison of error and measurement uncertainty mainly included instrument accuracy, sampling error and the accuracy of the analysis method: that is, all measuring instruments were calibrated, and their accuracy was between ±0.1% and ±1%. Through repeated sampling and analysis, the estimated sampling error was between ±2% and ±5%. By comparing with the standard method, the accuracy of the analytical method was within ±3%. The study first compared the predictive performance of four models for pH value and DO, as shown in Fig 11.

**Fig 12:**
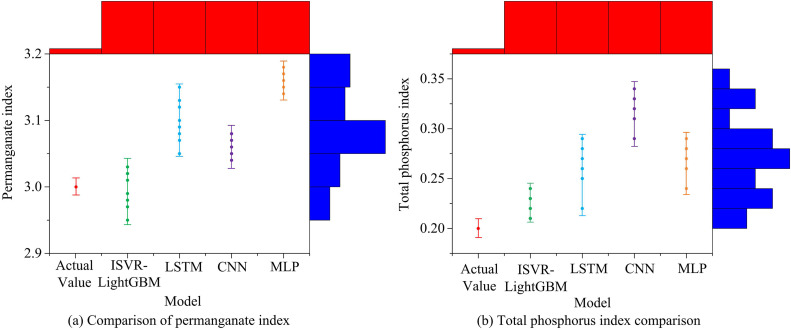
Comparison of the prediction effects of permanganate index and total phosphorus index.

Fig 11a shows a comparison of the performance of four models in predicting pH values. The outcomes revealed that after multiple predictions, the PV of the ISVR-LightGBM model tended to 7.18, which was very close to the AV of 7.2, indicating that the model had high accuracy in forecasting pH values. The pH predicted by the MLP model tended towards 7.25, with the largest difference from the AV, but it was also within an acceptable range. Fig 11b indicates a comparison of the predictive performance of four models for DO. The results indicated that the PV of ISVR-LightGBM tended towards 5.45 mg/L, which was nearly identical to the AV of 5.5 mg/L, and the group difference was small. The LSTM model had the worst prediction performance, with a PV of 5.6 mg/L, slightly higher than the ISVR-LightGBM model. The study further compared the predictive performance of four models for permanganate index and total phosphorus index, as shown in Fig 12.

**Fig 13:**
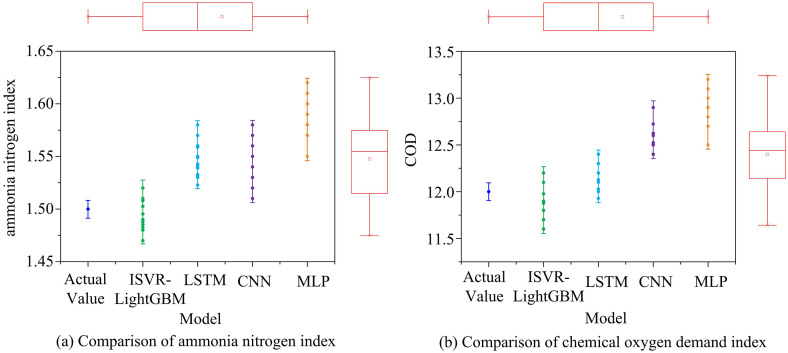
The prediction results of ammonia nitrogen index and chemical oxygen demand.

Fig 12a indicates a comparison of the predictive performance of four models for permanganate index. The results showed that after multiple predictions, the ISVR-LightGBM model tended towards a value of 2.99 mg/L, which was nearly akin to the AV of 3.0 mg/L, indicating that the model performed well in forecasting this indicator. The PV of the LSTM model tended to be 3.10, with significant intra group differences, and there were also significant differences between the PV and the AV. Fig 12b shows a comparison of four models for total phosphorus index. The results indicated that the PV of the ISVR-LightGBM model tended to be 0.22 mg/L, which was nearly akin to the AV of 0.2 mg/L, demonstrating high prediction accuracy. Meanwhile, the CNN model had the worst prediction performance for this indicator, with its prediction results tending towards 0.33 mg/L. The study then compared the prediction outcomes of four models for ammonia nitrogen index and chemical oxygen demand, as shown in Fig 13.

**Fig 14:**
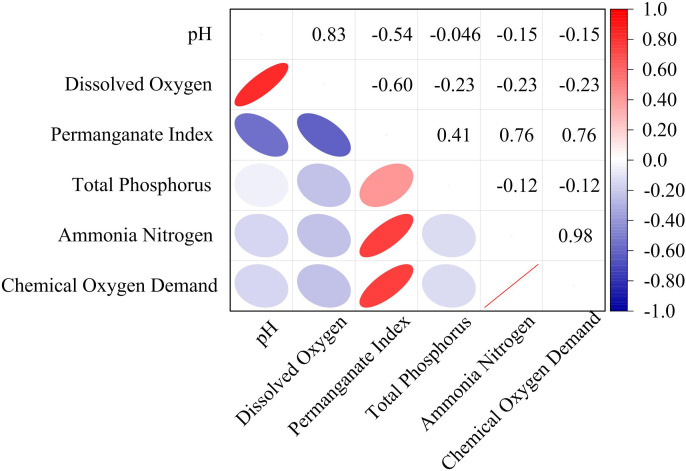
Results of correlation analysis.

Fig 13a shows a comparison of the prediction outcomes of four models for ammonia nitrogen index. The results showed that after multiple predictions, the ISVR-LightGBM model tended towards a value of 1.48 mg/L, which was very close to the AV of 1.5 mg/L. This indicated that the model had high accuracy in forecasting this indicator and was superior to other control models. The prediction results of CNN and MLP models tended to be 1.54 mg/L and 1.57 mg/L, respectively, while there were certain prediction differences within the group. Fig 13b shows a comparison of the prediction outcomes of four models for chemical oxygen demand. The results showed that after multiple measurements, the ISVR-LightGBM model tended to have a value of 11.8 mg/L, which was closest to the AV of 12.0 mg/L. The LSTM and CNN models followed closely in predicting the results, which tended to be 12.4 mg/L and 12.6 mg/L, respectively. At the end of the study, a correlation analysis was conducted on all the predicted indicators, and the results are shown in Fig 14.

In Fig 14, the pH value showed a moderate negative correlation (−0.60) with DO, indicating that when the pH value was high, the DO might be low. This value was consistent with the experimental results. Under alkaline conditions, the photosynthetic efficiency of aquatic plants might decrease, thereby reducing the production of DO. At the same time, it showed a strong positive correlation (0.83) with the permanganate index, indicating that when the pH value was higher, the permanganate index also tended to be higher. The permanganate index was moderately positively correlated with ammonia nitrogen (0.76). Meanwhile, it showed a moderately positive correlation (0.76) with chemical oxygen demand. Finally, ammonia nitrogen and chemical oxygen demand showed a strong positive correlation (0.98), indicating that their changing trends were highly consistent. When the ammonia nitrogen content was high, the chemical oxygen demand was also high. This result is consistent with the research result of H. Xia [48]. This report analyzed the relationships among ecosystem services, including synergy and trade-offs, and identified socio-ecological drivers. In the results of the correlation analysis, the correlation results of indicators such as chemical oxygen demand and dissolved oxygen are consistent with the research. To solve the problem of multicollinearity, the LightGBM algorithm could identify and select the features with the most predictive ability, while reducing the influence of multicollinearity among features. Through correlation analysis, features highly correlated with the target variable can be identified, which usually contribute the most to the model’s predictive ability and thus should be given priority in the feature selection process. Meanwhile, in time series analysis, correlation analysis could help identify the importance of features that change over time, thereby achieving dynamic feature selection.

## 5. Discussion

To efficiently and accurately diagnose the health status of rivers and lakes, an improved SMA optimized SVR model was studied, and a combined model was constructed by combining LightGBM feature selection, which was applied to intelligent diagnosis of river and lake ecosystems. In the experimental results, numerical results indicated that the optimized model improved in multiple key performance indicators: The RMSE was reduced to 0.031, the MAE was reduced to 0.021, and the R^2^ was improved to 0.942. The further optimized combination model had better prediction accuracy, with an RMSE of 0.022, an MAE of 0.016, and an R^2^ of 0.976, which aligned with the findings of L. Xu et al. L. Xu et al. proposed an ISVR prediction and sensitivity analysis model based on the current situation of predicting chlorophyll-a concentration in water and analyzing influencing factors. The findings demonstrated that the model could precisely forecast chlorophyll-a concentration and clarify the relative importance of various environmental parameters on chlorophyll-a concentration through sensitivity analysis [49]. Compared with the research, the method described in the study performed better in high-dimensional data, dynamic changing environments and resource-constrained environments. The ISVR-LightGBM model could adapt to the dynamic changes of water quality parameters and improve the prediction accuracy. In addition, the model also performed well in robustness indicators such as outlier sensitivity, noise resistance, and overfitting tendency, with an outlier sensitivity of 0.2, noise resistance of 0.8, and overfitting tendency of 0.1, significantly better than the control model. This result was slightly different from S. Gholizadeh’s. S. Gholizadeh et al. introduced a classification approach grounded in SVR kernel function for monitoring water changes in the eastern part of the Caspian Sea in Iran over the past decade. This method utilized Landsat-8 satellite data, combined with water index and SVR processing, to extract water areas and monitor changes. The results indicated that although the ISVR model had better performance indicators, its robustness was not as good as the improved MLP model [50]. Although this literature indicated that the improved MLP model might be superior to the SVR model in terms of robustness, the ISVR-LightGBM model proposed in the study achieved dual improvements in performance and robustness through feature selection, dynamic weights, and adaptive search mechanisms. The numerical results indicated that the model had high precision and dependability in predicting water quality parameters, as well as high robustness in handling data containing outliers and noise, which was particularly important for complex and variable river and lake ecosystems. The research results provided efficient and accurate methods for water quality prediction and ecosystem health diagnosis, effectively enhancing the ability to prevent and control ecological risks in rivers and lakes, and promoting the digital transformation of water environment management. In water quality monitoring, seasonal variations and long-term trends had a significant impact on water quality parameters. The ISVR-LightGBM model could capture these time-related changes by integrating advanced feature selection mechanisms and optimization algorithms. The LightGBM algorithm was used to identify the features that had the greatest impact on the prediction results, including those that changed significantly with the seasons, thereby improving the accuracy of the prediction.

In terms of model deployment, by leveraging the resources of the cloud service provider (AWS), computing power could be flexibly expanded, facilitating the deployment and management of the model. Additionally, the model was deployed on edge devices near the data collection point to reduce data transmission latency, which was suitable for application scenarios that require rapid response. During the real-time monitoring process, a medium-performance multi-core CPU was required, with at least 8GB of memory. Meanwhile, an SSD hard drive was needed to provide faster data read and write speeds, and a stable network was required to be capable of real-time data transmission and receiving prediction results. Based on the changes in data and business requirements, the model was set to be retrained once every quarter. When significant changes in data distribution are detected (such as seasonal changes or unexpected events), the retraining of the model will be directly triggered. For the prediction results, the feature importance analysis provided by LightGBM was utilized to explain which features had the greatest impact on the prediction results. It presents the decision-making process of the model through visualization tools, and at the same time provides specific case analyses to demonstrate how the model makes predictions based on input features. Meanwhile, in the actual deployment process, hardware costs include sensors, data collectors, and servers, etc. At the same time, the software requires customized data analysis software and regular maintenance of the system. In addition, energy costs need to be combined with local electricity prices, and the operation and maintenance team requires professional personnel, thereby generating additional labor costs. In water quality monitoring and ecosystem health diagnosis, the needs of stakeholders are diverse, including environmental regulatory agencies, water resource managers, public health departments and local communities. They need accurate and timely water quality prediction information to make decisions. However, operational limitations such as the challenges of data acquisition, the demand for computing resources, the complexity of model maintenance, and cost-benefit analysis are also factors that must be taken into account when implementing the model.

In addition, for different geographical regions, the model may perform best in specific geographical areas because water quality parameters may be affected by local climate, terrain and human activities. Therefore, data can be collected in various regions for training and validation, and a region-adaptive model version can be developed, which can automatically adjust parameters according to the characteristics of the region where it is located. Over time, changes in environmental conditions may lead to a decline in model performance. To address this issue, the model can be retrained or fine-tuned regularly using the latest data. In addition, the model can be designed to gradually update its parameters through online learning to adapt to new data. In addition, the model is highly sensitive to the quality of the input data. Noise and outliers in the data may affect the prediction results. Before the model is deployed, the data can undergo strict preprocessing, including cleaning, standardization and outlier handling. The training and prediction of the model may require relatively high computing resources, which may limit its application in resource-constrained environments. To reduce computing costs, it is possible to explore deploying models on edge devices to minimize reliance on central servers. Finally, the model may need to be trained for specific water quality parameters. Transferring to other parameters such as water temperature, conductivity, and cyanobacteria density may require additional adjustments. To enhance the universality of the model, a multi-task learning framework can be designed to predict multiple water quality parameters simultaneously. In addition, when the quality of the input data is poor and the features are not correlated, the performance of the model may not be good. The model may not be applicable when immediate decisions need to be made and there is not enough time to collect data. At the same time, the model is also not applicable when computing resources are limited.

In practical applications, datasets often contain missing values, which have a significant impact on the training and prediction performance of the model. First, the missing values in the data are detected through an automated script, and then the missing values are filled in based on the mean filling method. In the decision-making process of the model, the analysis of uncertainty is quite important. Confidence intervals can be set, and at the same time, the uncertainty of model parameters can be evaluated through resampling techniques, thereby obtaining the uncertainty estimation of the prediction results. The credibility of the prediction results depends on multiple factors, including data quality, model complexity and application scenarios. High-quality data can enhance the credibility of prediction results. If the data contains noise or bias, the prediction results may be unreliable. An uncertainty threshold can be set. When the prediction uncertainty exceeds the threshold, the prediction result should be treated with caution.

In summary, the intelligent diagnostic model based on improved SMA optimized SVR introduced in the research showed notable advantages in the health diagnosis of river and lake ecosystems. Through dynamic weighting strategy and adaptive search mechanism, the model improved its parameter optimization ability, solving the problems of high-dimensional data modeling and dynamic adaptability. The experimental findings validated the predictive accuracy of the model on multiple water quality indicators, including pH value, DO, permanganate index, total phosphorus, ammonia nitrogen, and chemical oxygen demand, all of which achieved high accuracy and robustness.

## 6. Conclusion

The research proposed an intelligent diagnosis model for river and lake ecosystems based on SVR, which effectively solved the problem of nonlinear coupling and complex dynamic evolution mechanism of water quality parameters in the health diagnosis of river and lake ecosystems. By introducing dynamic weight strategies and adaptive search mechanisms, the parameter optimization ability of the model has been significantly enhanced, enabling it to perform outstandingly in high-dimensional data modeling and dynamic adaptability. In addition, the combined model constructed by integrating LightGBM feature selection not only further optimizes the prediction accuracy but also effectively reduces the model complexity and computational cost, enhancing the practicality and efficiency of the model. In terms of robustness, the model demonstrates low sensitivity to outliers and high resistance to noisy data, while having a low tendency to overfit, which gives it an edge in complex and variable river and lake ecosystems. Despite obtaining favorable outcomes, the study still has certain limitations. The generalization ability of the model in different river and lake ecosystems needs further validation. Secondly, the computational efficiency of the algorithm and the flexibility of parameter adjustment still need to be improved. Future research directions should expand the applicability of the model, optimize algorithms to improve computational efficiency, and explore more feature selection methods to further enhance model performance.

## Supporting information

S1 File Minimal Data Set Definition. (DOC)
