# Supplementary material for: Intelligent diagnosis method for river and lake ecosystem health based on improved slime mold algorithm-optimized SVR
Source: PLoS One. 2026 Jan 21;21(1):e0340418. doi: 10.1371/journal.pone.0340418 (PMC12823004; doi:10.1371/journal.pone.0340418)
Supplement: S1 File — (DOC) [file pone.0340418.s001.doc]

**The data in Figure 5**

| Time window | RMSE | MAE | R² | Time delay error /h |
| --- | --- | --- | --- | --- |
| January | 0.023 | 0.015 | 0.975 | 0.51 |
| February | 0.025 | 0.017 | 0.973 | 0.62 |
| March | 0.022 | 0.014 | 0.977 | 0.38 |
| April | 0.024 | 0.016 | 0.974 | 0.49 |
| May | 0.026 | 0.018 | 0.972 | 0.70 |

**The data in Figure 6**

| Model | RMSE | MAE |
| --- | --- | --- |
| LightGBM (Default Settings) | 0.030 | 0.020 |
| ISVR-LightGBM | 0.022 | 0.016 |
| Mutual Information | 0.028 | 0.018 |
| PCA | 0.032 | 0.021 |

**The data in Figure 7(a)**

| Iteration | ISVR-LightGBM | LSTM | CNN | MLP |
| --- | --- | --- | --- | --- |
| 0 | 0.301 | 0.102 | 0.105 | 0.103 |
| 20 | 0.456 | 0.234 | 0.212 | 0.201 |
| 40 | 0.623 | 0.345 | 0.321 | 0.305 |
| 60 | 0.976 | 0.456 | 0.432 | 0.501 |
| 80 | 0.976 | 0.567 | 0.512 | 0.849 |
| 100 | 0.976 | 0.654 | 0.601 | 0.849 |
| 120 | 0.976 | 0.756 | 0.689 | 0.849 |
| 140 | 0.976 | 0.756 | 0.783 | 0.849 |
| 160 | 0.976 | 0.756 | 0.783 | 0.849 |
| 180 | 0.976 | 0.756 | 0.783 | 0.849 |
| 200 | 0.976 | 0.756 | 0.783 | 0.849 |

**The data in Figure 7(b)**

| Iteration | ISVR-LightGBM | LSTM | CNN | MLP |
| --- | --- | --- | --- | --- |
| 0 | 0.102 | 0.101 | 0.103 | 0.104 |
| 20 | 0.234 | 0.156 | 0.147 | 0.165 |
| 40 | 0.456 | 0.234 | 0.212 | 0.243 |
| 60 | 0.918 | 0.345 | 0.321 | 0.401 |
| 80 | 0.918 | 0.456 | 0.432 | 0.829 |
| 100 | 0.918 | 0.567 | 0.512 | 0.829 |
| 120 | 0.918 | 0.747 | 0.601 | 0.829 |
| 140 | 0.918 | 0.747 | 0.751 | 0.829 |
| 160 | 0.918 | 0.747 | 0.751 | 0.829 |
| 180 | 0.918 | 0.747 | 0.751 | 0.829 |
| 200 | 0.918 | 0.747 | 0.751 | 0.829 |

**The data in Figure 8**

| Parallel experiment | Model | Memory Usage (MB) | Peak Memory Usage (MB) |
| --- | --- | --- | --- |
| Parallel experiment 1 | ISVR-LightGBM | 120.5 | 180.4 |
|  | LSTM | 255.4 | 320.8 |
|  | CNN | 201.7 | 284.4 |
|  | MLP | 150.9 | 214.1 |
| Parallel experiment 2 | ISVR-LightGBM | 122.6 | 181.1 |
|  | LSTM | 253.3 | 321.7 |
|  | CNN | 201.8 | 283.2 |
|  | MLP | 151.5 | 213.3 |

**The data in Figure 9**

| Parallel experiment | Model | Outlier Sensitivity | Noise Resistance | Overfitting Tendency |
| --- | --- | --- | --- | --- |
| Parallel experiment 1 | ISVR-LightGBM | 0.19 | 0.80 | 0.08 |
|  | LSTM | 0.48 | 0.59 | 0.39 |
|  | CNN | 0.39 | 0.61 | 0.28 |
|  | MLP | 0.73 | 0.32 | 0.60 |
| Parallel experiment 2 | ISVR-LightGBM | 0.21 | 0.80 | 0.12 |
|  | LSTM | 0.53 | 0.62 | 0.38 |
|  | CNN | 0.42 | 0.62 | 0.33 |
|  | MLP | 0.64 | 0.28 | 0.51 |

**The data in Figure 10**

| Model | RMSE | MAE |
| --- | --- | --- |
| ISVR-LightGBM | 0.022 | 0.016 |
| SVR | 0.035 | 0.025 |
| SMA-SVR | 0.031 | 0.021 |
| ANN | 0.041 | 0.028 |
| DNN | 0.037 | 0.021 |
| CNN-GRU | 0.033 | 0.018 |

**The data in Figure 11**

| Model | pH | dissolved oxygen |
| --- | --- | --- |
| Actual Value | 7.20 | 5.5 |
| ISVR-LightGBM | 7.18 | 5.41 |
| ISVR-LightGBM | 7.17 | 5.41 |
| ISVR-LightGBM | 7.16 | 5.43 |
| ISVR-LightGBM | 7.16 | 5.42 |
| ISVR-LightGBM | 7.16 | 5.42 |
| ISVR-LightGBM | 7.17 | 5.43 |
| ISVR-LightGBM | 7.17 | 5.46 |
| ISVR-LightGBM | 7.18 | 5.47 |
| ISVR-LightGBM | 7.17 | 5.45 |
| ISVR-LightGBM | 7.17 | 5.47 |
| LSTM | 7.22 | 5.61 |
| LSTM | 7.23 | 5.62 |
| LSTM | 7.23 | 5.56 |
| LSTM | 7.23 | 5.59 |
| LSTM | 7.22 | 5.57 |
| LSTM | 7.23 | 5.60 |
| LSTM | 7.24 | 5.61 |
| LSTM | 7.22 | 5.62 |
| LSTM | 7.23 | 5.62 |
| LSTM | 7.23 | 5.61 |
| CNN | 7.19 | 5.57 |
| CNN | 7.18 | 5.50 |
| CNN | 7.21 | 5.52 |
| CNN | 7.22 | 5.53 |
| CNN | 7.24 | 5.54 |
| CNN | 7.23 | 5.55 |
| CNN | 7.22 | 5.57 |
| CNN | 7.22 | 5.58 |
| CNN | 7.19 | 5.58 |
| CNN | 7.20 | 5.54 |
| MLP | 7.22 | 5.52 |
| MLP | 7.23 | 5.53 |
| MLP | 7.22 | 5.53 |
| MLP | 7.26 | 5.44 |
| MLP | 7.24 | 5.44 |
| MLP | 7.25 | 5.48 |
| MLP | 7.22 | 5.47 |
| MLP | 7.25 | 5.48 |
| MLP | 7.25 | 5.47 |
| MLP | 7.24 | 5.46 |

**The data in Figure 12**

| Model | permanganate index | total phosphorus index |
| --- | --- | --- |
| Actual Value | 3.0 | 0.2 |
| ISVR-LightGBM | 3.02 | 0.21 |
| ISVR-LightGBM | 3.01 | 0.22 |
| ISVR-LightGBM | 2.99 | 0.22 |
| ISVR-LightGBM | 2.95 | 0.23 |
| ISVR-LightGBM | 2.97 | 0.24 |
| ISVR-LightGBM | 2.98 | 0.23 |
| ISVR-LightGBM | 3.03 | 0.21 |
| ISVR-LightGBM | 3.05 | 0.23 |
| ISVR-LightGBM | 3.05 | 0.21 |
| ISVR-LightGBM | 3.02 | 0.22 |
| LSTM | 3.1 | 0.22 |
| LSTM | 3.12 | 0.25 |
| LSTM | 3.13 | 0.25 |
| LSTM | 3.12 | 0.26 |
| LSTM | 3.08 | 0.27 |
| LSTM | 3.09 | 0.27 |
| LSTM | 3.07 | 0.28 |
| LSTM | 3.05 | 0.29 |
| LSTM | 3.09 | 0.25 |
| LSTM | 3.15 | 0.26 |
| CNN | 3.06 | 0.33 |
| CNN | 3.05 | 0.31 |
| CNN | 3.07 | 0.33 |
| CNN | 3.05 | 0.32 |
| CNN | 3.08 | 0.31 |
| CNN | 3.05 | 0.33 |
| CNN | 3.04 | 0.32 |
| CNN | 3.08 | 0.34 |
| CNN | 3.04 | 0.34 |
| CNN | 3.07 | 0.29 |
| MLP | 3.16 | 0.27 |
| MLP | 3.16 | 0.27 |
| MLP | 3.17 | 0.28 |
| MLP | 3.18 | 0.29 |
| MLP | 3.14 | 0.27 |
| MLP | 3.14 | 0.28 |
| MLP | 3.14 | 0.26 |
| MLP | 3.15 | 0.24 |
| MLP | 3.15 | 0.26 |
| MLP | 3.17 | 0.28 |

**The data in Figure 13**

| Model | ammonia nitrogen index | chemical oxygen demand |
| --- | --- | --- |
| Actual Value | 1.5 | 12 |
| ISVR-LightGBM | 1.48 | 11.8 |
| ISVR-LightGBM | 1.49 | 11.7 |
| ISVR-LightGBM | 1.48 | 11.8 |
| ISVR-LightGBM | 1.47 | 11.7 |
| ISVR-LightGBM | 1.51 | 11.9 |
| ISVR-LightGBM | 1.52 | 11.8 |
| ISVR-LightGBM | 1.48 | 11.6 |
| ISVR-LightGBM | 1.48 | 11.6 |
| ISVR-LightGBM | 1.48 | 12.1 |
| ISVR-LightGBM | 1.48 | 12.2 |
| LSTM | 1.55 | 12.2 |
| LSTM | 1.56 | 12.3 |
| LSTM | 1.54 | 12.3 |
| LSTM | 1.53 | 12.4 |
| LSTM | 1.55 | 12.1 |
| LSTM | 1.56 | 12.2 |
| LSTM | 1.57 | 12 |
| LSTM | 1.58 | 12.3 |
| LSTM | 1.56 | 12.3 |
| LSTM | 1.55 | 12.2 |
| CNN | 1.52 | 12.6 |
| CNN | 1.53 | 12.5 |
| CNN | 1.53 | 12.4 |
| CNN | 1.54 | 12.5 |
| CNN | 1.51 | 12.5 |
| CNN | 1.58 | 12.5 |
| CNN | 1.57 | 12.4 |
| CNN | 1.57 | 12.4 |
| CNN | 1.56 | 12.9 |
| CNN | 1.55 | 12.5 |
| MLP | 1.58 | 12.9 |
| MLP | 1.59 | 12.8 |
| MLP | 1.6 | 12.7 |
| MLP | 1.61 | 12.7 |
| MLP | 1.62 | 12.5 |
| MLP | 1.61 | 12.9 |
| MLP | 1.57 | 13 |
| MLP | 1.55 | 13.2 |
| MLP | 1.58 | 13.2 |
| MLP | 1.57 | 13.1 |
